# Nurse Training in War-Time

**Published:** 1916-07-08

**Authors:** 


### THE EDITOR'S LETTER-BOX.

### Nurse Training in War-Time.

*To the Editor of* The Hospital.

Sir,—In reply to a paragraph in your issue of June 24, I should like to say that the disadvantages of training in war-time commented upon therein had occurred to, and been lamented by, me.

The only suggestions that occur to me as remedies or palliatives are :—

(1) An appeal should be made by matrons to experienced sisters to remain in civilian hospitals, or at any rate to give only one year's service under the military authorities. (Any trained nurse can cope with the duties in a military hospital, but only a sister of some years' standing can satisfactorily train probationers.)

(2) Third-year probationers should be placed in charge of one ward, while a sister is placed in charge of two, over the staff nurses.

(3) As few probationers as are necessary for the keeping an adequate staff should be admitted for training. (The profession will, shortly after the war, be crowded with trained nurses released from military work; there need therefore be no fear of a shortage of trained workers.)

(4) Probationers trained during the war should be induced to stay on for six months after the completion of their third year. They should rank as senior nurses, and be given every opportunity for responsible work under good teachers, in order to make up arrears. (It is presumed the war cannot last much more than two years longer. Therefore probationers who commenced their training in 1914 might have the six months' final tuition under some sisters released from military service. Those who entered hospital in 1915 and 1916 will have even a better chance, as it is to be hoped that peace will have been declared before their training is over.)

With everything now tending to raise the status of nurses, it would be deplorable if those who will be launched in the profession after the war should fail—owing to lack of adequate training—to reach even as high a standard as those of the present generation.—Yours faithfully, Sister Norton.

